# Gender Differences for Health Indicators in a Sample of School Dropout Adolescents: A Pilot Study

**DOI:** 10.3390/ijerph19137852

**Published:** 2022-06-26

**Authors:** Francesca Mastorci, Maria Francesca Lodovica Lazzeri, Paolo Piaggi, Cristina Doveri, Anselmo Casu, Gabriele Trivellini, Irene Marinaro, Andrea Bardelli, Alessandro Pingitore

**Affiliations:** 1 Clinical Physiology Institute, CNR, Via Moruzzi, 1, 56124 Pisa, Italy; mastorcif@ifc.cnr.it (F.M.); mariafrancesca.lazzeri@ifc.cnr.it (M.F.L.L.); cristina.doveri@ifc.cnr.it (C.D.); webmaster@easywebmaster.eu (A.C.); gabriele.trivellini@ifc.cnr.it (G.T.); irene@ifc.cnr.it (I.M.); andreab.bardelli@gmail.com (A.B.); 2 Department of Information Engineering, University of Pisa, 56126 Pisa, Italy; paolo.piaggi@gmail.com

**Keywords:** health-related quality of life, risk factors, school dropout, substance use, adolescence

## Abstract

*Background*: The ever-increasing prevalence of school dropout (SD) highlights the need to gain insight into risk factors for dropout causes and consequences. The aim of this study was to evaluate the gender differences for health indicators in a sample of school dropout adolescents. *Methods*: Data were collected regarding 450 adolescent’s SD (19 ± 2 years; 308 males), and health-related quality-of-life (HRQoL) and risk behaviors were assessed by means of a standardized questionnaire. *Results:* The results revealed that the female population was characterized by a compromised health indicator profile in terms of both risk behaviors and HRQoL dimensions. *Conclusion:* These findings indicate that SD is a multidimensional phenomenon, for which the implementation of multiple educational, social, and psychological policies aimed at mitigating the issue are recommended.

## 1. Introduction

School dropout is defined as leaving an educational system without obtaining a minimal qualification, in most cases a higher secondary education diploma [1]. In the last decade, nearly 1.7 million of the more than 6 million students enrolled in the first year of high school did not make it to their senior year. Although estimates of dropout rates seem to be higher in developing countries, with particular focus on West Asia (43%) and Sub-Saharan Africa (36%), recent evidence suggests that even developed countries such as those in Europe have increasing numbers of dropout rates, with a peak in Spain (22%) and a greater incidence among males [2]. The latest data in Italy indicate that the dropout rate in 2019 was 3.82%, with a higher prevalence in vocational schools [3]. Although it is often difficult to differentiate causes from consequences, it is probable that school dropout results from the many changes that occur during adolescence in physical, cognitive, emotional and social dimensions. These modifications create instability, leading adolescents toward dysfunctional and risky behaviors. The most common of these behaviors are the use of alcohol and other illicit substances such as tobacco, internet and social media use/abuse, and unprotected sex [4], as well as dysfunctional eating behaviors and a lack or an excess of physical activity. Additionally, both internalizing and externalizing disorders have relationships with school dropout; among externalizing ones, disruptive behavior seems to prevent educational processes [5], while anxiety and depression are among the most encountered internalizing problems [6,7]. Much research has suggested that these disruptive behaviors can create a vicious cycle by compromising social relationships with peers, family, and school, which then leads to school absenteeism and dropout [8]. However, quantifying dropout rates is not easy because the assessment criteria are often not standardized, although reducing the school dropout rate below 10% is one of the goals currently confirmed by the European Strategy [9,10]. In this context, in order to implement any policies aimed at preventing, reducing and characterizing the dropout phenomenon, it is also important to evaluate any gender differences in both risk behaviors and the perception of well-being related to school dropout. Generally, given the different socialization of girls and boys, female adolescents have poorer perceptions of their own health, lower levels of life satisfaction in the psychosocial context, some variation in emotional experiences, and an increased frequency of somatic symptoms than males [11]; however, to our knowledge, there have been no studies assessing gender differences in terms of health and risk behaviors variables among school dropout adolescents. 

Although one third of psychopathologies of adulthood originate during adolescence, there are no data correlating this susceptibility with school dropout, let alone whether there are gender differences. Furthermore, it is important to emphasize that well-being is a complex and integrated framework of variables including psychosocial, emotional, and risk behaviors. An integrated framework of well-being could allow for the identification of strong and the fragile characteristics of each adolescent to potentiate the former and change or improve the latter through the application of personalized educational programs.

Therefore, the objective of this study was to evaluate the gender differences for health indicators, intended for use as risk behavior and health-related quality-of-life dimensions, in a sample of school dropout adolescents in order to highlight a more preventive approach for the containment and prevention of the dropout phenomenon from the earliest stages of development. 

## 2. Materials and Methods

### 2.1. Study Population

Data regarding school dropout were collected as part of the AVATAR (“A new purpose for promotion and eVAluation of healTh and well-being Among healthy teenageRs”) project. The AVATAR project is aimed at developing a new tool to assess lifestyle habits, social context, emotional status, and mental skills in adolescents, as well as to define an integrated index of the best indicators of well-being [12]. In total, 680 boys and girls, aged between 18 and 21 years, were included in this study. School dropout adolescents were enrolled in collaboration with the regional agency FORMATICA according to the following inclusion criteria: absence of neuropsychiatric or other diseases, signed informed consent, and completion of the entire questionnaire.

Of 680, 230 were excluded for the following reasons: diagnosed neuropsychiatric or other diseases (n = 20), absence of sign informed consent (n = 75), and incomplete questionnaires (n = 135). Therefore, the final study population consisted of 450 adolescents (19 ± 2 years; 308 males). Participants were previously instructed on how to fill out the questionnaires and complete the tests. All tests were performed during participants’ computer lessons during school time. No incentive was provided to adolescents or parents. A research assistant was available to provide information and technical support to complete questionnaires. 

### 2.2. Data Collection

Data were collected with the AVATAR Web-tool [12]. A socio-demographic data record was used to collect information on gender, age, and schooling. The Italian version of KIDSCREEN-52 was used to assess health-related quality of life [13,14]. KIDSCREEN is a self-report questionnaire designed to address health-related quality of life (HRQoL), which can be used to monitor and measure the personal experiences in children and adolescents about their perception of health status and well-being. The questionnaire, which describes physical, psychological, mental, social, and functional aspects of well-being, consists of 52 items grouped in 10 dimensions [13,14]. KIDSCREEN questionnaires were psychometrically tested using data obtained in a multicenter European study that included a sample of 22,827 children recruited from 13 countries [15]. Substance use and abuse (cannabis, cocaine, heroin, inhalants, amphetamines, etc.) were assessed as present if participant reported having used substances one or more times a week. Experiences in the use of social media over the past year were evaluated with the Bergen Social Media Addiction Scale (BSMAS) [16], which contains six items reflecting core addiction elements (i.e., salience, mood modification, tolerance, withdrawal, conflict, and relapse). Eating attitudes and behaviors were assessed using EAT-26, which consists of three subscales: dieting, eating preoccupation, and oral control [17].

### 2.3. AVATAR Approach: Psychological Well-Being Index

The AVATAR approach focuses on the integration of three components of health-related well-being—lifestyle habits (LH), emotional status (ES), and social context (SC)—as perceived by adolescents [18]. The three components were set from the different variables analyzed by questionnaires according to a structural model previously described by Mastorci and colleagues [19].

In detail, the path analysis technique was used in this study to measure the extent to which a model fits a dataset and allowed for the testing of interrelationships between several variables simultaneously. Confirmatory factor analysis was used to test an overall measurement model that included five correlated latent variables. The overall model fit was assessed using different statistics. First, a chi-square analysis was used. The other indices were the root mean square error of approximation (RMSEA) (values between 0.05 and 0.08 indicate an acceptable fit, and values < 0.05 indicate a good fit), comparative fit index (CFI) (values > 0.90 indicate a reasonable fit, values >0.95 indicate a good fit), and standardized root mean square residual (SRMR) (values < 0.10 indicate a good fit). The measurement model was first tested to ensure that each of the observed variables was a sufficient indicator of the hypothesized latent variables.

From the sum of the three components, we generated a personalized well-being index (PWBI) ranging from 0 to 100 based on the AVATAR model, as reported previously [19]. 

### 2.4. Statistical Analysis

Statistical data analyses were performed using SPSS software. Data are presented as mean ± SD, and categorical variables are presented as counts and percentages. The Shapiro–Wilk test was used to assess the normality of data distribution. A *p*-value ≤ 0.05 was considered statistically significant. Changes in HRQoL and PWBI by gender were analyzed by Student’s unpaired t-test. The χ^2^ test was used for continuous and categorical variables.

## 3. Results

### 3.1. Socio-Demographic Characteristics and Risk Behaviors of Study Population by Gender

Table 1 shows the sample demographic characteristics and risk behaviors. In total, 450 participants (32% girls; mean age: 19.38 ± 1.89) were included in the analyses. Age was similar between male and female participants (male: 19.35 ± 1.69 vs. female: 19.46 ± 2.28; *p* = ns). Regarding substance use and abuse, expressed both as presence and frequency, no statistically significant differences were observed for alcoholic drinks, nicotine, cannabis, cocaine, and other illegal substances. Girls made more use of psychotropic drugs in terms of frequency in the last month (*p* < 0.05). The addictive use of social media was described more in the female population compared to their male counterparts (BSMAS: 4.2% vs. 0.4%, respectively; *p* < 0.01; Table 2), as was the presence of eating disorders (EAT-26: 14% vs. 5.8%, respectively; *p* < 0.01). Regarding the sexuality dimension, females reported more risk behaviors related to the non-use of contraceptive methods than males (55.6% vs. 44.4%, respectively; *p* < 0.05; Table 2).

### 3.2. Gender Differences on Health-Related Quality of Life and Personalized Well-Being Index

Descriptive data of health-related quality of life in our sample separated by gender are presented in Table 3. All variables were normally distributed according to the Shapiro–Wilk test. Regarding HRQOL, several variables significantly differed according to gender.

Males perceived a higher physical and psychological well-being (*p* < 0.001), emotional background (*p* < 0.001), perception of self (*p* < 0.001), and autonomy (*p* < 0.05) compared to the female population. There were also gender differences regarding social context, particularly in the family environment, where girls reported significantly lower well-being (*p* < 0.001) and more episodes of bullyism (*p* < 0.05) than males.

**Figure 1:**
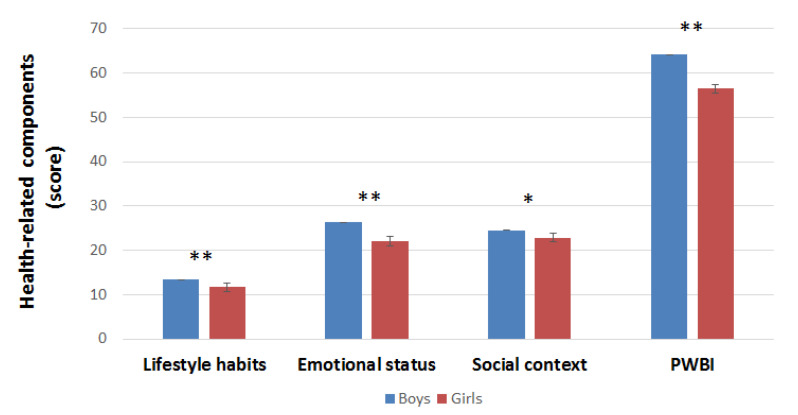
Gender differences in Psychological Well-Being Index (PWBI) and its components. Data given as mean ± SD (95% CI). * *p* < 0.05; ** *p* < 0.001.

Regarding health-related well-being components integrated into the PWBI (Figure 1), lifestyle habits (*p* < 0.001), social context (*p* < 0.05), and emotional status (*p* < 0.001), as well as the overall PWBI (*p* < 0.001), showed significantly lower scores in the female population than the male population. 

## 4. Discussion

In the present paper, we describe preliminary results about gender differences for health indicators, intended as both risk behaviors and health-related quality-of-life dimensions, in a sample of school dropout adolescents in order to highlight a preventive approach for the containment and prevention of dropout at an early stage of development. According a more comprehensive framework to prevent dropout, as well as protect the general health and well-being of adolescents, preventive strategies should be more oriented to improve psychosocial and environmental conditions with potential health implications through the development of networks of different institutional, political, and social health stakeholders.

We also describe the development and application of a multidimensional and personalized index for this category of adolescents. A ground-breaking characteristic of this index is its integrated feature, because the analysis is based on the relationships among the different weights of the variables belonging to the three dimensions, i.e., lifestyle, emotional status, and social context, as well as the role of risk behavior in the modulation of them. This index, which includes subjective information about health status based on individual perceptions of health including social and psychological perspectives, was already developed for a different class of adolescents (10–14 years) by Mastorci and colleagues; we enriched this index with risk behaviors in order to understand how and if they interfere with well-being in order to develop potential preventive strategies [19,20].

The main health indicator results, by gender, can be summarized according to three observation levels: risk behaviors, health-related quality-of-life dimensions, and psychological well-being index. Overall, we can say that girls showed a worsening of health indicators at all levels of analysis. Firstly, substance use and risk behaviors were predominantly found in the female population in terms of the frequency of psychotropic drug use, unprotected sexual activity, social addiction, and eating disorders. This is probably linked to a gender effect that emerged for HRQoL, i.e., girls were shown to have a lower well-being perception in terms of the psychological and physical well-being, mood/emotion, self-perception, and parent relationship dimensions. Our results are in line with what has been highlighted by previous works regarding adolescents in general, not dropout adolescents specifically, as female adolescents have been shown to have a poorer perception of their own health, a lower level of life satisfaction in the psychosocial context, and an increased frequency of somatic symptoms compared to male adolescents [11].

Additionally, when we analyzed the three health-related well-being components forming the PWBI, we found that female school dropouts exhibited reduced well-being scores, both in total and in the single areas constituting the total.

Although late adolescence (our target population) is the period of greatest psychophysical well-being, it is also a period when dysfunctional behaviors may begin, resulting in increasing future susceptibility to diseases. In fact, it is estimated that about half of the mental health problems in adulthood arise during adolescence [21]. There are also important neurological changes, chief among them the process of “pruning” that can increase the brain’s efficiency but also expose it more to risk [22]. This biological phenomenon is responsible for the hyperactivity of the dopaminergic system that then translates into risky behaviors by reducing one’s ability to assess their potential harmfulness, resulting in the need to develop preventive approaches and strategies [23,24]. So, there is a neurobiological cause for adolescents’ attraction to the discovery of emotions and immediate pleasures. Adolescents’ vulnerability to risk behaviors is supported by changes not only in their brain structure but also in several neurotransmission systems, including the dopaminergic, serotonergic, noradrenergic and glutamatergic systems. For example, the increased activity of the dopaminergic system tends to inhibit the prefrontal cortex such that critical risk assessment skills are reduced and one is more exposed to impulsive behavior, such as experimenting with illegal drugs [22].

To our knowledge, most studies on school dropout have been focused on retrospective adult samples. Although possible mechanisms linking substance use with school dropout are unclear, the relationship between substance abuse and school dropout is among the most studied [5], and it has been suggested that students who are involved in drug or alcohol abuse are more likely to drop out from school. Another important dimension that longitudinally implicated in school dropout is the social context, both family and school-related [1]. From the social perspective, school performance and the home environment are strictly connected, probably mediated by socio-economic status or family structure, and especially involved in the socialization process [25]. 

In this context, to prevent mental health problems, especially in the long term, the support of parental education, families, and peer groups are crucial in overall adolescence, although previous studies have mainly focused on the early adolescent period. Some research has shown that healthy lifestyle behaviors are associated with better family functioning [26]. On the other hand, poor family conditions are linked to an increased risk for unhealthy behaviors with consequences such as obesity [27]. Additionally, adolescents with a parent who smokes have a greater chance of developing poor diet quality [28]. Additionally, the emotional dimension and social settings, regarding family or peers, are strictly interconnected and facilitate adaptive behavior [29]. 

Regarding school factors, absenteeism has been identified as a risk factor for school dropout, resulting in negative developmental outcomes such as deviant behaviors in some cases [30]. From a psychological point of view, the findings for this study agree with previous evidence also described by our group demonstrating that girls have a lower well-being perception and higher levels of depression and anxiety than boys [18,19,31], although we have found no evidence of a gender difference in psychotropic drug use, other than a trend towards greater use in girls.

The reasons for these results, already reported in other studies, may be related to differences in emotional processing in addition to intrinsic biological traits [32]. Adolescence is a time of behavioral divergence between boys and girls, probably due to different hormonal developments, although it is difficult to base this difference on objective parameters [33]. For example, studying the differences in health and wellness in adolescence has made it possible to understand the results of different attitudes toward internalizing and externalizing responses to the environment. Another possible reason for these differences could be related to the social context, as studies have shown how the exposure of girls to social stressors can create alterations in the neural reward circuitry and determine not only depression in adolescence but also a greater predisposition to depression in adulthood, as well as a lower ability to respond to stress in a resilient way [34].

Additionally, it may be necessary to consider the impact of the fact that girls are diagnosed with depression three times more often than boys on the use of digital media, assuming addictive behavior. Although previous evidence is scarce and conflicting, our results, which demonstrated an addictive use of social media in female population, are in accordance with data suggesting that digital media use might have more impact on girls’ well-being than boys’ [35]. In this context, as also evidenced by our findings showing that female adolescents exhibited more eating disorders, it should be noted that social media use is linked to worries about body weight among adolescent girls [36]. This may be because girls probably spend more time than boys crafting their online image as a result of more self-objectification, placing more emphasis on how their physical bodies appear to others [37]. 

Unsurprisingly, the use of social media accounts has been shown to be associated with eating disorders; in particular, a previous study demonstrated that female adolescents with Snapchat, Facebook, and Instagram profiles were significantly likely to have eating disorders and body weight alterations [38]. This higher prevalence of eating disorders in girls is also consistent with our previous research on a younger sample (early adolescence) in which we demonstrated a close relationship between weight status categories and HRQoL that was more pronounced in females than in males, showing that weight status affects the psychological dimension more than other dimensions [39].

## 5. Limitations and Future Directions

Despite the strengths of our analyses, including the possibility of using our results to enhance adolescents’ aptitude skills (empowerment) and adaptive response to stimuli (resilience), which are intermediate steps for achieving psychophysical well-being and counter educational poverty, certain limitations warrant discussion.

First, the sample consisted of 450 dropout adolescents between the ages of 18 and 21 who predominately lived in Northern Italy, so they not representative of the overall dropout phenomenon in Italy. Subsequent studies on a larger and more representative populations will be necessary to confirm our preliminary results.

Additionally, the relatively small sample size may have limited our power to detect smaller effect sizes, especially those related to adolescent risk behaviors and HRQOL categories. In addition, since our study was aimed to assess gender differences in risk behaviors and psychosocial profiles, we asked no questions about confounding variables such as family structure and any other pre-existing issues that may have induced school dropout. Finally, the roles of parents’ educational styles, families, and peer groups were only studied in general terms based on the KIDSCREEN test, which we used to evaluate these dimensions in adolescents. 

Future research should examine whether our findings can be generalized to different age groups using a larger sample size and should aim to understand how the variables underlying dropout affect each other. Moreover, our assessments were all based on self-reporting, so future work should include the use of validated instruments that provide more comprehensive subdimensions for previous experience.

## 6. Conclusions

The preliminary findings of this study demonstrate that health indicators in a sample of dropout adolescents showed gender differences, with the female population reporting greater impairment in psychosocial dimensions correlated with well-being and risk behaviors. Our results, although collected with a pilot sample not representative of the whole dropout phenomenon, are the first to our knowledge related to this category of adolescents. 

Identifying a profile that combines both risk behaviors and everything related to health-related quality of life may help in the prevention of disease in adulthood. This is mainly because this susceptible period, which may leave most adolescents with a positive sense of self and a good quality of life, is also a sensitive time when many forms of psychopathology may begin to manifest.

In addition, monitoring long-term outcomes could be helpful in understanding the dropout phenomenon even better. From more general perspective, in order to clarify the contribution of the psychosocial profile to alterations in well-being and health-related quality of life, as well as its association with drug use in dropout adolescents, researchers also need to define a more comprehensive preventive approach. In order to change unhealthy behaviors, it is necessary to create conditions that favor healthy lifestyles through a transversal approach to risk factors and social settings from childhood to late adolescence. The innovation of the AVATAR-Dropout framework resides in the development of an integrated and holistic approach to well-being that incorporates different variables, including psychosocial, emotional, and risk behavior, into the Psychological Well-Being Index, which provides the opportunity to assess the contribution of each component in relation to each other for the definition of well-being perception and, thus, to identify dimensions that need to be reinforced and changed through the application of focused preventive programs. 

Therefore, fostering the well-being of adolescents must be prioritized to bridge the gap between psychosocial determinants and school dropout from both the health and education perspectives (as well as the addiction field), with the ultimate aim of better centering health and well-being around adolescents’ needs and fragility.

## Figures and Tables

**Table 1:** Substance use and abuse in total population and by gender.

Variables			Total (*n* = 450)	Boys (*n* = 308)	Girls (*n* = 142)	*p*-Value
**Nicotine**		Yes	218 (48)	149 (48)	69 (49)	ns
		Not	192 (43)	133 (43)	59 (42)	
	Frequency (die)	0	172 (38)	119 (39)	53 (37)	ns
		1–9	144 (32)	98 (32)	46 (32)	
		10–30	69 (15)	44 (14)	25 (18)	
		Over 40	2 (0)	2 (1)	0 (0)	
**Cannabis**		Yes	76 (17)	58 (19)	18 (13)	ns
		Not	320 (71)	211 (69)	109 (77)	
	Frequency (last month)	0	294 (65)	197 (64)	97 (68)	ns
		1–9	41 (9)	32 (10)	9 (6)	
		10–30	18 (4)	13 (4)	5 (4)	
		Over 40	14 (3)	11 (4)	3 (2)	
**Illegal Drug Use**		Yes	10 (2)	6 (2)	4 (3)	ns
		Not	388 (86)	264 (96)	124 (87)	
	Frequency (last month)	0	332 (74)	225 (73)	107 (75)	ns
		1–9	7 (2)	3 (1)	4 (3)	
		10–30	2 (0)	1 (0)	1 (1)	
		Over 40	2 (0)	2 (1)	0 (0)	
**Psychotropic Drugs**		Yes	22 (5)	11 (4)	11 (8)	ns
		Not	376 (84)	259 (84)	117 (82)	
	Frequency (last month)	0	332 (74)	229 (74)	103 (73)	0.05
		1–9	5 (1)	1 (0)	4 (3)	
		10–30	10 (2)	4 (1)	6 (4)	
		Over 40	3 (1)	2 (1)	1 (1)	
**Alcoholic Drinks**		Yes	177 (39)	125 (41)	52 (37)	ns
		Not	227 (50)	151 (49)	76 (54)	
	Frequency (last month)	0	248 (55)	174 (56)	74 (52)	ns
		1–9	121 (27)	78 (25)	43 (30)	
		10–30	10 (2)	8 (3)	2 (1)	
		Over 40	3 (1)	2 (1)	1 (1)	

Data are expressed as number (%). ns: not significant. *p* was calculated with the χ^2^ test.

**Table 2:** Risk behaviors in total population and by gender.

Variables		Total (*n* = 450)	Boys (*n* = 308)	Girls (*n* = 142)	*p*-Value
**Sexual Behavior**	Sexual activity	250 (56)	179 (58)	71 (50)	ns
	No Sexual activity	172 (38)	110 (36)	62 (44)	
	Contraceptive use	211 (69)	155 (50)	56 (39)	0.05
	No Contraceptive use	194 (43)	124 (40)	70 (49)	
**Bsmas**	AUSM	6 (1)	1 (0)	5 (4)	0.01
	Not AUSM	376 (84)	262 (85)	114 (80)	
**Eat-26**	ED risk	33 (7)	16 (5)	17 (12)	0.01
	ED no risk	364 (81)	260 (84)	104 (73)	

Data are expressed as number (%). BSMAS: Bergen Social Media Addiction Scale; EAT-26: Eating Attitudes Test; AUSM: addictive use of social media; ED: eating disorders; ns: not significant. *p* was calculated with the χ^2^ test.

**Table 3:** Gender differences in KIDSCREEN-52 domains.

VARIABLES	Boys (*n* = 308)	Girls (*n* = 142)	*p*-Value
**Physical well-being**	44.1 ± 8.42	38.95 ± 7.7	<0.001
**Psychological well-being**	43.88 ± 9.02	40.25 ± 8.43	<0.001
**Mood/Emotion**	45.43 ± 8.86	40.41 ± 9.26	<0.001
**Self-perception**	48.86 ± 9.74	42.74 ± 8.31	<0.001
**Autonomy**	47.7 ± 8.1	45.62 ± 9.12	<0.05
**Parent relationship**	46.32 ± 9.65	42.07 ± 10.59	<0.001
**Financial resources**	47.03 ± 9.55	45.86 ± 9.07	=0.225
**Peers**	49.38 ± 10.8	47.84 ± 11.02	=0.166
**School environment**	47.03 ± 7.56	47.08 ± 7.01	=0.950
**Social acceptance (Bullyism)**	49.73 ± 10.05	47.13 ± 11.64	<0.05

Data given as mean ± SD (95% CI). Data on the KIDSCREEN-52 dimension were calculated as the mean T-scores according to KIDSCREEN test. *p*-values were calculated with Student’s unpaired t-test.

## Data Availability

The datasets used and/or analyzed during the current study are available from the corresponding author on reasonable request.
